# Siah1 proteins enhance radiosensitivity of human breast cancer cells

**DOI:** 10.1186/1471-2407-10-403

**Published:** 2010-08-03

**Authors:** Hai-Tao He, Emmanouil Fokas, An You, Rita Engenhart-Cabillic, Han-Xiang An

**Affiliations:** 1 Department of Radiotherapy and Radiation Oncology, Philipps-University Marburg, Baldingerstr. D-35043 Marburg, Germany

## Abstract

**Background:**

Siah proteins play an important role in cancer progression. We evaluated the effect of Siah1, its splice variants Siah1L and the Siah1 mutant with the RING finger deleted (Siah1ΔR) on radiosensitization of human breast cancer cells.

**Methods:**

The status of Siah1 and Siah1L was analysed in five breast cancer cell lines. To establish stable cells, SKBR3 cells were transfected with Siah1, Siah-1L and Siah1ΔR. Siah1 function was suppressed by siRNA in MCF-7 cells. The impact of Siah1 overexpression and silencing on apoptosis, proliferation, survival, invasion ability and DNA repair was assessed in SKBR3 and MCF-7 cells, also in regards to radiation.

**Results:**

Siah1 and Siah1L mRNA expression was absent in four of five breast cancer cells lines analysed. Overexpression of Siah1 and Siah1L enhanced radiation-induced apoptosis in stable transfected SKBR3 cells, while Siah1ΔR failed to show this effect. In addition, Siah1 and Siah1L significantly reduced cell clonogenic survival and proliferation. Siah1L sensitization enhancement ratio values were over 1.5 and 4.0 for clonogenic survival and proliferation, respectively, pointing to a highly cooperative and potentially synergistic fashion with radiation. Siah1 or Siah1L significantly reduced invasion ability of SKBR3 and suppressed Tcf/Lef factor activity. Importantly, Siah1 siRNA demonstrated opposite effects in MCF-7 cells. Siah1 and Siah1L overexpression resulted in inhibition of DNA repair as inferred by increased levels of DNA double-strand breaks in irradiated SKBR3 cells.

**Conclusion:**

Our results reveal for the first time how overexpression of Siah1L and Siah1 can determine radiosensitivity of breast cancer cells. These findings suggest that development of drugs augmenting Siah1 and Siah1L activity could be a novel approach in improving tumor cell kill.

## Background

Breast cancer is the most common malignancy and the major cause of cancer-related deaths of women in industrialized countries [1]. Radiotherapy consists one of the cornerstones in the treatment of patients with breast cancer and its role has been extensively studied during the last decades [2,3]. Clinical studies have demonstrated a major benefit of adjuvant radiotherapy in increasing disease-free survival and overall survival. A profound impact of ionizing radiation in improving local control and reducing disease recurrence has also been shown in patients undergoing breast-conserving therapy [3]. However, radiotherapy is associated with side effects including an increased risk of cardiovascular disease [4]. Finding agents that sensitize malignant cells to radiation would therefore increase tumor response while minimizing toxicity to surrounding organs by lowering effective therapeutic doses.

Regulation of protein stability through the ubiquitin-proteasome pathway is now being recognized as a major mechanism of regulating a diverse array of cellular processes [5]. The Drosophila seven in absentia (Sina) protein and its human homolog Siah (seven in absentia homolog) are members of an evolutionarily highly conserved family of E3 ubiquitin ligases [6]. The members of this family (Siah1 and Siah2) contain a N-terminal RING domain that binds E2 proteins, followed by two zinc-finger domains involved in protein-protein interactions [6-8]. Siah ligases regulate the ubiquitination and proteasomal degradation of several proteins including DCC, β-catenin, c-Myb, alpha-synuclein, the CDK activator RINGO and BAG-1, a Hsp70/Hsc70-binding protein, suggesting a role for Siah proteins in the regulation of cell proliferation, migration, apoptosis and tumor suppression [7-17]. Recent studies demonstrated that lower expression of Siah2 was associated with resistance to endocrine therapy in breast cancer [18]. Furthermore, Siah2 expression predicated a favourable clinical outcome of breast cancer patients [18,19]. Siah1 expression is upregulated by p53, revealing a link between genotoxic injury and destruction of β-catenin [9,10,20] and reduced T-cell factor/lymphoid enhancer factor (Tcf/Lef) activity [11,18]. Furthermore, several Siah1 splicing variants such Siah1L and Siah1 S involved in the degradation of β-catenin degradation have been previously described [21,22]. A recent study demonstrated a framework of ATM/ATR and Siah1 through the stabilization of HIPK2, a mediator of DNA-damage induced apoptosis, implicating Siah1 in DNA damage response [22,23].

Although Siah1 presents an attractive target for cancer therapy, its potential radiosensitizing effects have not been previously studied. In search for novel strategies to enhance radiosensitivity of breast cancer, we investigated the role of Siah1 and its related variant Siah1L on the radiation response of SKBR3 and MCF-7 breast cancer cells using different approaches. Furthermore, we analysed the impact of Siah1 overexpression on the biologic behaviour of breast cancer cells by employing invasion and Tcf/Lef reporter studies.

## Methods

### Plasmid Construction and Transfection

Siah1, Siah-1L and the Siah1 mutant with the RING finger deleted that expresses Siah1ΔR were gifts from Prof. SI Matsuzawa, the Burnham Institute, USA [20]. The Tcf/Lef-responsive luciferase reporter gene (Topflash), the negative control with mutated Tcf/Lef binding site (Fopflash), and the *Renilla *luciferase reporter plasmid (pRL-TK) as an internal control were obtained from Upstate Biotechnology, USA. The human breast cancer cell lines BT-20, MCF7, MD-MBA231, SKBR3 and ZR75-1 were obtained from the American Type Culture Collection (Manassas, VA). To establish stable cell lines, SKBR3 cells (1.5 × 10^5^) were grown on 60 mm^2 ^culture dish containing RPMI-1640 medium supplemented with 10% FCS, 1% glutamine, 1% penicillin and streptomycin (Biochrom-Seromed, Germany) at 37°C one day before transfection. Siah1, Siah-1L or Siah1ΔR plasmids were transfected into cells using the FuGENE 6 transfection reagent (Roche, Mannheim, Germany) according to the manufacturer's protocol. Cells were allowed to rest for about 12 to 18 h after transfection, and the medium was changed with 800 μg/mL of G418 (Invitrogen) for 2 weeks. Stable clones were selected for G418 resistance and screened for Sial1 protein level with immunoblotting.

The small interfering RNA (siRNA) duplexes were designed and purchased from Qiagen (Cambridge, MA, USA). The sequences for the Siah1 siRNA were: Siah1 siRNA 5'-AACTCCTGCCTCCTTATGTATTT-3'. Siah1 siRNA-2, 5'-GAUAGGAACACGCAAGCAA-3'; Siah1 siRNA-3, 5'-GUUGCAUGUAGUAACACUA-3'. A control siRNA was used as well (no silencing): 5'-AAGAGCCGTCAGACTGCTACA-3'. Roti-Fectplus was used fro the transient transfections with SIAH1 siRNA, or control siRNA, according to the manufacturer's instructions (Carlroth, Karlsruhe, Germany).

### Immunoblotting

Cells were washed in PBS and lysed in NP40 buffer containing 50 mM Tris-HCl, 1 mM PMSF, 150 mM NaCl, 1% NP-40 and protease inhibitor cocktail (Roche Diagnostics). After determination of the amount of protein in the cell lysates, samples were then resolved by polyacrylamide gel (PAGE) and electrophoretically blotted onto polyvinylidene difluoride membranes as described previously [20]. Membranes were incubated with anti-FLAG-M5 monoclonal antibody (1:500, Sigma Science, Hamburg, Germany). After incubation with a horseradish-conjugated goat anti mouse antibody, the protein content was visualized using enhanced chemiluminescence (Amersham, Freiburg, Germany). Siah1 and Siah2 protein expression was analysed as well using anti-Siah1 (YZ-12, Santa Cruz, CA, USA) and anti-Siah2 (N-14, Santa Cruz, CA, USA) primary antibodies, respectively together with the appropriate secondary antibodies. α-Tubulin monoclonal antibody was used as a control (1:500, Santa Cruz, CA, USA).

### Irradiation (IR)

Single dose of 0, 2, 4, 6 or 8 Gy with a rate of 650 cGy/min was given using an Elekta Synergy linear accelerator with 6 MV-photons, at room temperature.

### Reverse transcription-PCR

Total RNA was extracted from cultured cells and collected at different time points after IR using RNEasy protect mini kit (Qiagen, Germany), according to manufacturer's instructions. cDNA synthesis was performed in a 20 μl reaction volume with 0.5 μg of total RNA using cDNA synthesis kit (Fermentas, USA). The mixture was incubated at 37°C for 1 h followed by 10 min in 70°C for inactivation of reverse transcriptase. The primer sequences for amplification of Siah1 and Siah1L were described previously [21]. The primer sequences for amplification of Siah1ΔR and glyceraldehyde-3-phosphate dehydrogenase (GAPDH) were as follow: Siah1ΔR forward primer ACCTCGAAGTGTCCACCATC and reverse primer ACTGCATCATCACCCAGTCA (product size, 547 bp); GAPDH forward primer TGGTCACCAGGGCTGCTT and reverse primer AGCTTCCCGTTCTCAGCCTT (product size, 150 bp). PCR was done using a MyiQ detection system (Bio-Rad). The PCR conditions were 95°C for 5 min followed by 35 cycles of denaturation at 95°C for 30 seconds, annealing at 60°C for 30 seconds, and extension at 72°C for 1 min.

### Apoptosis and clonogenic survival assay

Transfected SKBR3 cells as well as MCF-7 after siRNA for Siah1, including a control siRNA, were plated into 100 mm^2 ^dishes and were irradiated (4 Gy). Apoptosis was analyzed 24 h after IR by flow cytometry using Annexin V-FITC apoptosis detection kit (Sigma Aldrich, Munich, Germany). Staining was performed at indicated time points after IR according to the manufacturer's instructions, and flow cytometry was conducted on a flow cytometer (BD FACScan). Statistical analysis was performed using WinMDI V2.9 software. Survival curves were obtained by means of standard colony formation assay. Transfected SKBR3 and MCF-7 cells were irradiated (1 × 10^3^) and plated onto 25-mL culture flash for 80 to 100 colonies per flash. After 14 days of incubation, colonies were fixed with 10% formalin and stained with crystal violet. Colonies with >50 cells were scored as a surviving colony. Surviving fraction was calculated as: (mean colonies counted)/(cells plated) × (plating efficiency), where plating efficiency was defined as (mean colonies counted)/(cells plated) for unirradiated controls. Experiments were conducted in triplicate and are presented as means ± standard deviation (SD) from three independent experiments. All survival fractions (SF) were fitted into the linear quadratic model. The ID_10 _and SD for survival curves in SKBR3 cells were determined at 10% cell survival. The sensitizer enhancement ratio (SER) in SKBR3 cells was defined as SER = mean inactivation dose (radiotherapy)/mean inactivation dose (plasmid +radiotherapy), as previously described. SER>1 indicate radiosensitization [24-26].

### Proliferation Assay

Transfected SKBR3 cells as wells as MCF-7 cells, upon Siah1 siRNA, were seeded at a density of 1 × 10^4 ^cells/well in a 96-well microplate and grown for 6 h and subsequently exposed to 0, 4 and 8 Gy IR. After 24 h of incubation at 37°C, the cell proliferation reagent WST-1 was added (10 μl/well) in SKBR3 cells for 30 min, according to the manufacturer's instructions (Roche, UK). The reaction product was quantified by measuring the absorbance using an ELISA reader (HTS 7000, Perkin-Elmer, Rodgau, Germany) and Software HT-Soft (Perkin-Elmer, USA). The experiments were performed three times in triplicate and presented as means (± SD). Cell viability curves were fitted using the linear quadratic model and the dose resulting in 50% of cell growth inhibition (IC_50_) was calculated, as previously described [25,26]. The cell viability of MCF-7 cells was measured at 2, 4 and 6 days using the same method, as described above. Cell viability was normalized to the untreated group. A control siRNA group was included as well.

### Immunofluorescence

Stable transfected SKBR3 cells (5000) were seeded on cover slips and incubated for 12 h and either unirradiated or irradiated with X-ray (4 Gy). At 3 and 6 h after IR, cells were fixed in cold methanol (-20°C) for 5 min, permeabilized with 1% Triton/PBS for 10 min and then blocked in PBS containing 0.3% Triton X-100, 3% BSA for 1 h. Fixed, permeabilized cells were incubated with in 100 μL mouse monoclonal anti-γH2AX antibody at 1:100 dilution (Upstate Biotechnology, Waltham, MA) for 30 min at room temperature. This was followed by incubation with Alexa 488 conjugated goat anti-mouse secondary antibody (Molecular Probes, Eugene, OR, 1:200 dilutions) for 1 h at room temperature. Finally, cells were counterstained with 4',6-Diamidino-2-phenylindole (DAPI, 0.1 μg/mL). The sections were mounted with fluorescent mounting medium (Dako, Germany). The fluorescent images were examined on an Olympus JMT-2 photomicroscope (Hamburg, Germany). For each treatment condition, γH2AX foci were determined in at least 100 cells.

### Invasion assay

In vitro cellular invasion was determined the ability of cells to invade a synthetic basement membrane (BD Biosciences, Heidelberg, Germany). Stably transfected SKBR3 cells (2 × 10^4^) and MCF-7 cells (5 × 10^4^), upon Siah1 siRNA, were diluted in 350 μl medium and transferred into precoated Matrigel membrane filters (8 μm pore size). The inserts were placed in the bottom chamber containing RPMI 1640 medium supplemented with 5% FCS. Following 48 h incubation at 37°C, filters were fixed in 100% methanol for 15 min, air dried, and stained with haematoxylin stain for 2 min. Noninvading cells on the upper surface were removed with a cotton swab, whereas invading cells on the underside of the filter were counted using an inverted microscope. All experiments were done in triplicate and a minimum of 10 individual fields at ×10 magnification per filter was counted.

### Luciferase reporter assay

Luciferase reporter assays were done at least in triplicate as described [20]. SKBR3 cells (4 × 10^4^) as well as MCF-7 cells (4 × 10^4^), after siRNA, were grown on 24-well tissue culture plates. In each well, transient DNA transfection was carried out with 50 ng DNA of the Tcf/Lef-responsive luciferase reporter gene (Topflash) or a negative control (Fopflash), 5 ng of the *Renilla *luciferase reporter plasmid (pRL-TK) as an internal control for the normalization of transfection efficiency and 50 ng of the indicated expression plasmids or an empty control vector. After 24 h, the luciferase and *Renilla *activity was measured using the dual luciferase assay system (Promega, Mannheim) and the luciferase activity was normalized against the *R*enilla luciferase activity and are presented as means ± SD from three independent experiments. Together with the Siah1 siRNA group, a control siRNA was tested as well in MCF-7 cells.

### Statistical analysis

Quantitative data were expressed as means ± SD. The significance of differences between the means was assessed using one-way ANOVA or student *t*-test using SPSS 15.0 software. Statistically significant difference was considered as *P *< 0.05.

## Results

### Siah1 gain-of-function in SKBR3 cells and silencing in MCF-7 breast cancer cells

**Figure 1:**
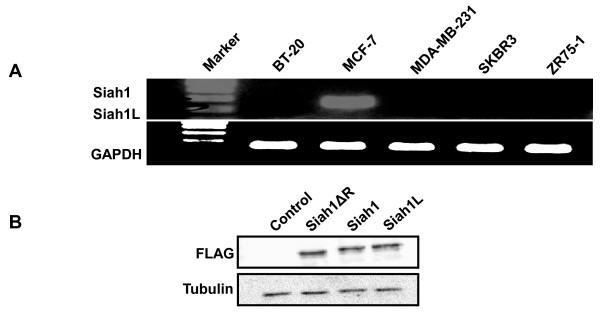
**Expression of Siah1 and Siah2 in SKBR3 cells**. A: All but the MCF-7 breast cancer cells lacked expression of Siah1 and Siah1L. mRNA expression was determined by RT-PCR. GAPDH was used as an internal control. B: Ectopic expression of Siah1, Sial1L or Siah1ΔR in SKBR3 cells. Cells were stable transfected with plasmids encoding FLAG- tagged Siah1, Sial1L or Siah1ΔR proteins. Protein level was determined by Immunoblot analysis with anti FLAG antibody.

**Figure 2:**
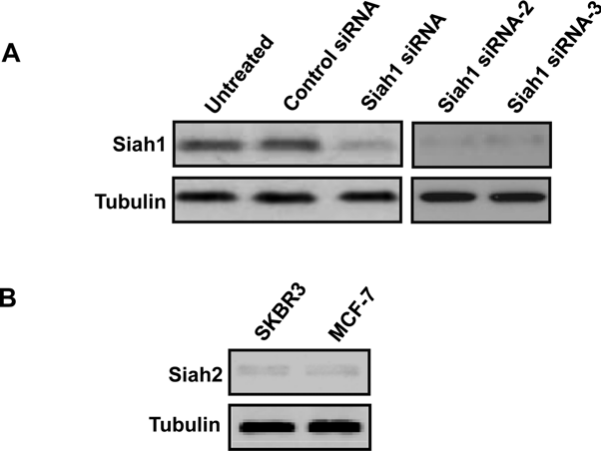
**Knockdown of Siah1 expression in MCF7 cells**. A: Western blot showed that siRNA as well as siRNA-2 and siRNA-3 for Siah1 resulted in suppressed protein expression in MCF-7 cells. B: Siah2 expression is decreased in SKBR3 and MCF-7 cells. α-Tubulin was used as an internal control.

The expression of Siah1 and its splice variant Siah1L in breast cancer cell lines was determined using RT-PCR with specific primers recognizing the common sequences of both siah1 and siah1L. In line with previous report [10,27], MCF7 breast cancer cells exhibited strong expression of Siah1 and Siah1L, whereas BT-20, MDA-MB-231, SKBR3 and ZR75-1 breast cancer lines lacked any expression of Siah1 and Siah1L analysed (Figure 1A). The SKBR3 cells without detectable endogenous expression of Siah1 and Sial1L were chosen to establish stable gene expression. Stable clones were selected for G418 resistance and screened for relative equivalent levels of exogenous expression of Siah constructs detected by using immunoblotting (Figure 1B). Additionally, we performed siRNA for Siah1 in MCF-7 to further investigate its role in radiosensitization. Western blot confirmed blockade of Siah1 in MCF-7. Off-target effects can occur during siRNA [28] and therefore two additional Siah1 siRNAs (siRNA-2 and siRNA-3) were tested as well. They also confirmed blockade of Siah1 protein in MCF-7 cells. Control siRNA was used as well (Figure 2A). Siah2 protein expression was absent in both SKBR-3 and MCF-7 cells (Figure 2B), as previously demonstrated [29].

### Siah1 determines apoptosis and survival of SKBR3 and MCF-7 breast cancer cells in response to IR

**Figure 3:**
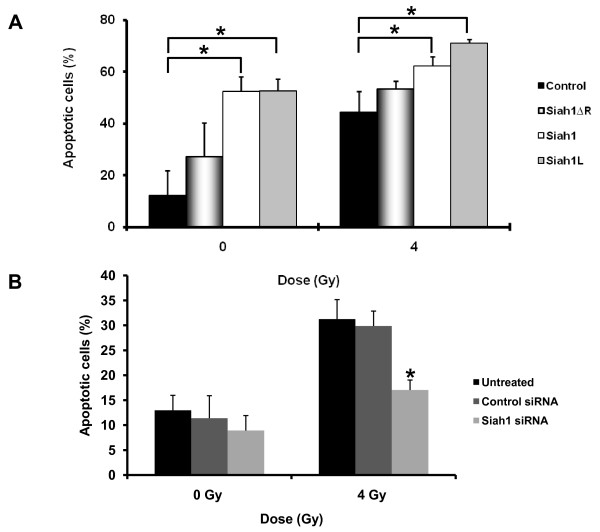
**Effect of Siah1 gain-of-function and silencing in IR-induced apoptosis**. SKBR3 cells were transfected with indicated plasmids and irradiated with 0 and 4 Gy IR. Apoptosis was determined at 24 h after IR by FACS analysis. Columns, mean of three independent experiments; bars, SD; *, P < 0.05. Siah1 functions were silenced by siRNA. A control siRNA was used as well. Apoptosis was determined at 24 h after IR by FACS analysis. Columns, mean of three independent experiments; bars, SD; *, Siah1 siRNA vs. both untreated and control siRNA at 4 Gy, P < 0.05.

**Figure 4:**
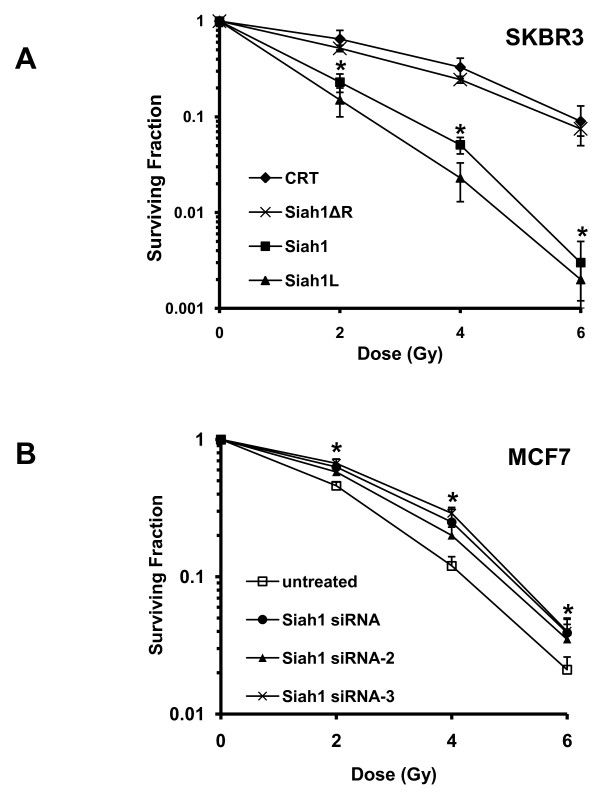
**Effect of Siah1 on the clonogenic survival of SKBR3 and MCF-7 cells after IR**. Cells transfected with indicated plasmids were irradiated with a single dose of 2, 4 or 6 Gy. Unirradiated cells (0 Gy) were used as control. A: Siah1 and more potently Siah1L decreased clonogenic survival in both irradiated and control cells. Colonies greater than 50 cells were counted and clonogenic survival was determined by crystal violet staining 14 days after IR. Lines, mean of three independent experiments; bars, SD; *, P < 0.05. B: siRNA for Siah1 resulted in reduced radiosensitivity of MCF-7 cells in response to IR. Silencing of Siah1 by siRNA-2 and siRNA-3 revealed results similar to the main Siah1 siRNA. Lines, mean of three independent experiments; bars, SD; *, P < 0.05.

Whether Siah1 may have an effect on radiation response has not yet been addressed. We therefore investigated the effects of Siah1 and its splice variants on breast cancer cells following IR. We found that overexpression of Siah1 and Siah1L significantly enhanced IR-induced apoptosis in SKBR3 cells compared with the control transfected with empty vector while Siah1 ΔR showed a small but non-significant difference (Figure 3A, control, 44.5 ± 7.9; Siah1ΔR, 53.4.2 ± 2.9; Siah1, 62.3 ± 3.5; Siah1L, 70.9 ± 1.5). Notably, Siah1 and Siah1L increased apoptosis also in unirradiated group (p < 0.05; control, 12.2 ± 9.5; Siah1ΔR, 27.2 ± 13.0; Siah1, 52.3 ± 5.6; Siah1L, 52.5 ± 4.5). Importantly, siRNA for Siah1 resulted in reduced apoptosis in response to IR as compared to the untreated group (Figure 3B, untreated, 31.27 ± 3.9; control siRNA 29.87 ± 4.5; Siah1 siRNA, 17.04 ± 2.1) while in unirradiated group no difference was observed (Fig 3B). To further analyze the radiosensitizing ability of Siah1 and Siah1L, we used clonogenic assay to assess survival of stably-transfected cells after IR. Importantly, overexpression of Siah1L and Siah1 cooperated with IR to reduce clonogenic growth of SKBR3, as compared with irradiated control cells (p < 0.05; Figure 4A). As illustrated in Table 1, a SER_10 _of 1.87 was observed with Siah1L gain-of-function, indicating a highly cooperative and potentially synergistic fashion between Siah1L and IR. Siah1 induced a rather additive than synergistic radiosensitizing effect (SER_10 _of 1.26) while no significant difference in survival was noted with Siah1ΔR (Table 1). In addition, Siah1 and Siah1L reduced clonogenic survival also in unirradiated cells, as compared with the control group. Loss-of-function for Siah1 by siRNA demonstrated a significant reduction in the radiosensitivity of MCF-7 cells, even at a dose of 2 Gy, as assessed by clonogenic survival (Figure 4B; p < 0.05). To exclude the possibility of "off-target" effects [28], we tested the impact of Siah1 silencing in radiosensitivity of MCF-7 cells by using two additional siRNAs (Siah1 siRNA-2 and Siah1 siRNA-3). The latter revealed results similar to the main Siah1 siRNA used (Figure 4B), whereby a significant decrease (p < 0.05) in radiosensitivity was detected.

**Table 1:** Effect of the expression of Siah genes and radiation on cell survival

	**IC**_**10 **_**± SD (Gy)**	**SER**_**10**_
**Control**	5,8 ± 0,3	1.00
**Siah1**	4,6 ± 0,1	1.26
**Siah1L **	3,1 ± 0,2	1.87
**SiahΔR**	5,6 ± 0,2	1.03

IC_10_: Irradiation dose at 10% of cell survival; SD: Standard deviation; SER_10_: Sensitizer enhancement ratio at 10% cell survival.

### Siah1 regulates viability in SKBR3 and MCF-7 breast cancer cells

**Figure 5:**
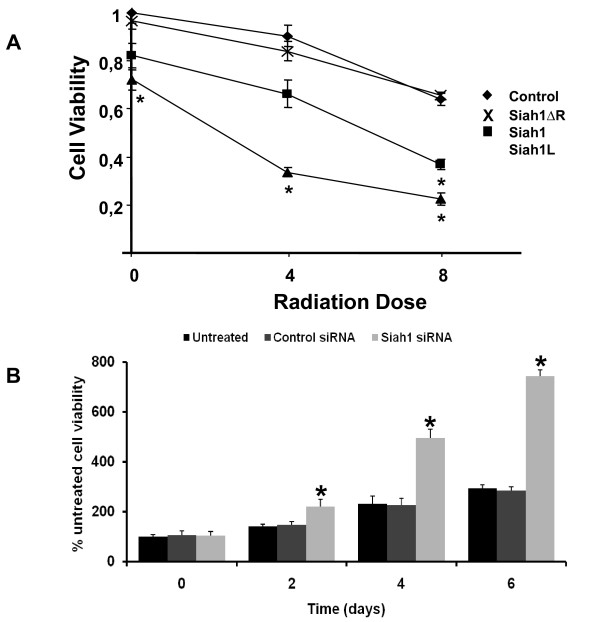
**Effect of Siah1 on SKBR3 and MCF-7 cell viability**. Metabolic activity was assessed by WST assay on SKBR3 cells transfected with indicated plasmids and escalating doses of IR (0, 4 and 8 Gy) as well as in MCF-7 cells after siRNA for Siah1. A: Cell viability was significantly reduced upon Siah1 and Siah1L transfection, both in irradiated and control cells. The WST assay was done 24 h after IR and normalized to unirradiated control. Lines, mean of three independent experiments; bars, SD; *, P < 0.05. B; Siah1 siRNA potently increased cell viability in MCF-7 cells at the different time points tested. Columns, mean of three independent experiments; bars, SD; *, Siah1 siRNA vs. both untreated and control siRNA, P < 0.05.

To determine the effect of Siah1 on the cell viability of SKBR3 cells after IR, SKBR3 cells were irradiated with different single doses (0, 4 or 8 Gy) and incubated for 24 h. The overexpression of Siah1 and Siah1L resulted in a significant dose-dependent reduction of the proliferation of unirradiated as well as irradiated cells, as compared with control vector (Figure 5A, p < 0.05). Potentially synergistic enhancement of radiocytotoxicity with a SER_50 _of 4.65 for cell viability was observed when Siah1L was combined with IR (Table 2). The proliferation assay indicated that the expression of Siah1 also results in increased sensitivity to ionizing radiation but less potently. As shown, Siah1ΔR exhibited no effect with a SER_50 _of 0.99 (Table 2). Moreover, Siah1 siRNA revealed an increase in MCF-7 cell viability at 2, 4 and 6 days upon suppression of Siah1 activity (Figure 5B; p < 0.05). Control siRNA was used as well.

**Table 2:** Effect of the expression of Siah genes and radiation on cell viability

	**IC**_**50 **_**± SD (Gy)**	**SER**_**10**_
**Control**	9,3 ± 0,3	1.00
**Siah1**	6,6 ± 0,2	1.41
**Siah1L **	2,0 ± 0,2	4.65
**Siah1ΔR**	9,4 ± 0,3	0.99

IC_50_: Irradiation dose at 50% of cell viability inhibition; SD: Standard deviation; SER_10_: Sensitizer enhancement ratio at 10% cell survival.

### Siah1 and Siah1L enhance γH2AX foci in SKBR3 cells after IR exposure

**Figure 6:**
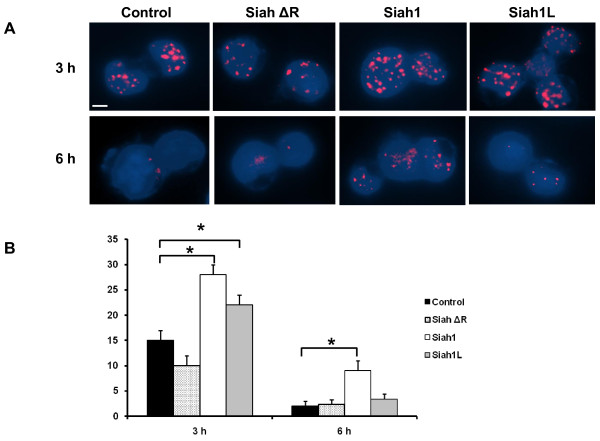
**Influence of Siah1 on DNA damage in response to IR**. A: transfected SKBR3 cells were irradiated with a single dose of 4 Gy followed by immunofluorescence at the 3 and 6 h after IR. Unirradiated cells were used as control. Siah1L significantly increased γH2AX foci at 3 h and the latter were more persistent and numerous 6 h post-IR. Quantitative analysis of γH2AX foci per cell averaged over 100 cells per data point. Columns, mean; bars, SE; *, P < 0.01. B: Representative merged images of DAPI (blue) and γH2AX (red) at 3 h and 6 h post-IR are shown. Scale bars, 10 μm.

To gain insight into the molecular mechanisms of radiosensitization of Siah and to investigate its effect on the initial DNA damage response to IR we monitored the fate of double-strand breaks (DSBs) in stable transfected cells after IR (4 Gy) using γH2AX, a landmark marker of DSBs [30,31]. Induction of γH2AX foci was significantly increased in Siah1- and Siah1L-transfected cells at 3 h post-IR compared with cells transfected with empty vector (p < 0.01; Figure 6A). Furthermore, Siah1L prolonged the persistence of γH2AX foci at relatively high levels at 6 h post-IR as compared with control cells, suggesting inhibition of DNA repair (p < 0.01; Figure 6A and 6B).

### Siah1 modifies tumor cells invasion in SKBR3 and MCF-7 breast cancer cells

**Figure 7:**
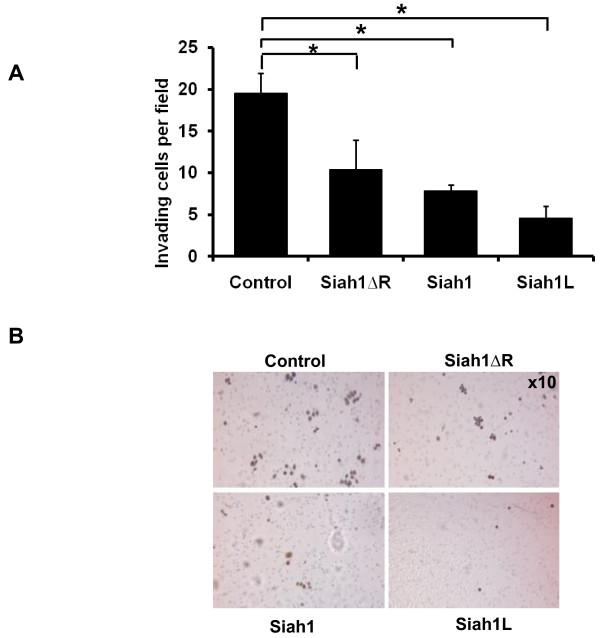
**Effects of Siah1 expression on invasion of SKBR3 cells**. A: The invasion of SKBR3 cells was studied in a modified Boyden chamber assay. A significant decrease in invasion was detected in transfected SKBR3 cells relative to control group. Cell numbers were counted in 10 individual high-powered fields. Columns, mean; bars, SD; *, p < 0.05. B: Representative photomicrographs Magnification ×20.

In an effort to define the significance of expression of Siah1 on the biological behaviour of breast cancer, we performed cell invasion study with a modified Boyden chamber assay. Siah1 and Siah1L gain-of-function in SKBR3 cells, upon transfection, significantly reduced the ability of these cells to invade Matrigel compared with control vector (p < 0.05; Figure 7A). Siah1ΔR transfection displayed a less potent but still significant effect (control, 19.5 ± 2.3; Siah1ΔR, 14.4 ± 3.5; Siah1, 7.8 ± 0.7; Siah1L, 4.6 ± 1.4); (Figure 7A and 7B).

**Figure 8:**
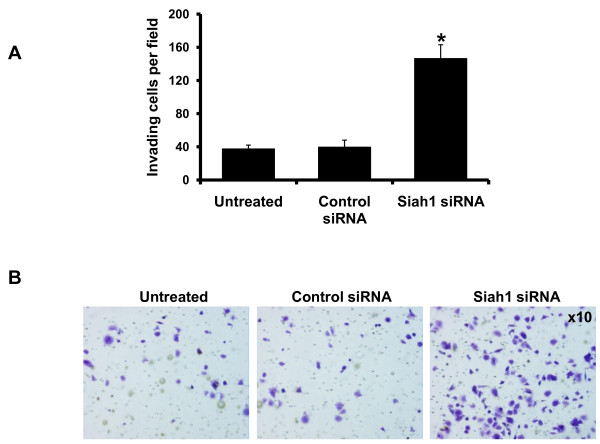
**Effects of Siah1 knockdown on invasion of MCF-7 cells**. C: Siah1 siRNA resulted in significant increase in MCF-7 cell invasion. D: Cell numbers were counted in 10 individual high-powered fields. Columns, mean; bars, SD; *, p < 0.05, Siah1 siRNA vs. both untreated and control siRNA.

Siah1 siRNA displayed a significant increase of the number of invading MCF-7 cells, as compared with the untreated and the control siRNA group (untreated, 38 ± 4; control siRNA, 40 ± 8; Siah1 siRNA, 147 ± 16) (p < 0.05; Figure 8A and 8B).

### Siah1 mediates Tcf/Lef activity in SKBR3 and MCF-7 breast cancer cells

**Figure 9:**
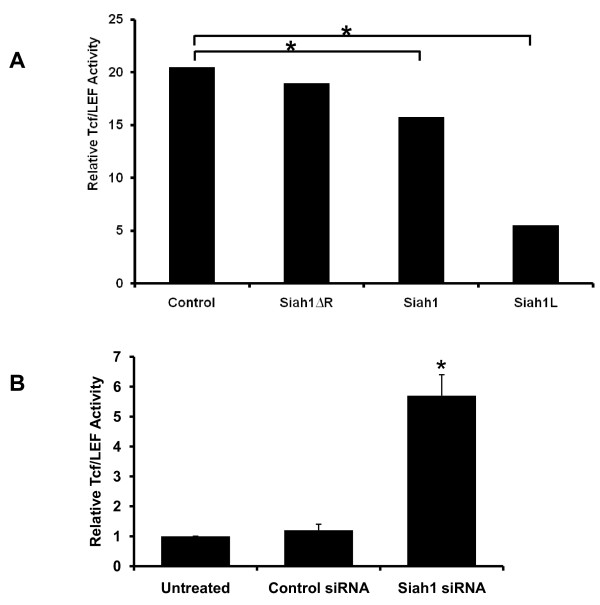
**Effects of Siah1 on Tcf/Lef regulated transcription activity in SKBR3 and MCF-7 cells**. A: Transfected cells were cotransfected with Topflash or Fopflash plasmid, the Renilla luciferase reporter plasmid (pRL-TK) as an internal control and the indicated expression plasmids. Luciferase activity was measured at 24 h after transfection and plotted after normalizing with respect to the Renilla luciferase activity. Each experiment was performed at least three times. Columns, mean; bars, SD; *, p < 0.05. B; Functional inhibition of Siah-1 ubiquitin-ligase by siRNA increased TCF/Lef transcriptional activity in MCF-7 cells at 24 hours after transfection. Each experiment was performed three times. Columns, mean; bars, SD; *, Siah1 siRNA vs. both untreated and control siRNA p < 0.05.

Previous studies have demonstrated the role of Siah-1 in degradation of β-catenin and in reduction of activity of Tcf/Lef transcription factor [11,13]. To examine the effect of Siah1 and Siah1L on Tcf/Lef activity in SKBR3 cells, we performed a luciferase reporter assay. In this assay, a reporter plasmid with a mutated Tcf/Lef-binding site was used as negative control. Siah1L gain-of-function significantly decreased Tcf/Lef activity while Siah1 showed a lower but significant effect, as compared with control SKBR3 cells (p < 0.05; Figure 9A). Tcf/Lef activity was not affected by overexpression of Siah1ΔR (control, 20.5 ± 2.2; Siah1, 15.7 ± 0.9; Siah1L, 5.5 ± 2.9; Siah1ΔR, 19.0 ± 1.6) (p > 0.05; Figure 9A). As expected, Siah1 siRNA demonstrated increased Tcf/Lef activity in MCF-7 cells, as assessed by the luciferase assay (Figure 9B, p < 0.05).

## Discussion

Due to intrinsic resistance of many tumors to established therapies, including radiotherapy, current attempts to improve the survival of cancer patients largely depend on strategies to increase tumor cell sensitivity. Siah1 has been studied for use in the therapy of human breast cancer and a number of different genetically-engineered Siah1 variants have been constructed in an effort to enhance chemotherapy efficacy [10,22,32].

In recent years, the proteasome pathways have been searched for the development of new targeted therapies and chemosensitizers in cancer therapy [33]. However, their potential to increase the therapeutic efficacy of radiation therapy and/or to reduce radiation-mediated side effects remains largely unknown. In the present study, we investigated whether Siah1 also plays a direct role in mediating radiation response. We found that gain-of-function for Siah1, and especially its splice variant Siah1L, sensitized SKBR3 breast cancer cells to the cytotoxic effects of IR while siRNA for Siah1 decreased radiosensitivity in MCF-7 breast cancer cells. We showed that overexpression of Siah1L resulted in a significantly increased rate of spontaneous and radiation-induced apoptosis and decreased SKBR3 cell viability. Accordingly, Siah1 siRNA exerted opposite effects in regards to apoptosis and cell viability in MCF7-cells. In addition, Siah1 increased DNA double-strand breaks induced by IR in SKBR3 cells, and finally resulted in an increased radiosensitivity as determined by the clonogenic survival assay. These effects were more pronounced after overexpression of Siah1L. Similarly, Siah1 siRNA revealed enhanced clonogenic survival of MCF-7 cells in response to IR. Our data support the potentially synergistic effect of the Siah1L modalities. In vitro experiments using proliferation and clonogenic assays showed synergistic cytotoxicity in SKBR3 breast cancer cells. These findings highlight the notion that susceptibility of human cancer to cytotoxic therapies critically depends on intact proteasomal-ubiquitin degradation pathways [32,33]. Weber et al. have previously showed that the proteasome inhibitor bortezomib can sensitize head and neck cancer cells to IR [34]. Moreover, Cao et al. revealed that bortezomib combined with docetaxel sensitized Bcl-2-overexpressing human prostate cancer cells to IR [35]. Furthermore, Celastrol, a proteasome inhibitor, sensitized prostate cancer cells to IR, both in vitro and in vivo, by impairing DNA damage processing and augmenting apoptosis [36]. Thus, targeting ubiquitin-proteasome pathway targets, such as Siah1 and Siah1L, may significantly alter treatment response

Another major finding of this study is that transfection with Siah1 and Siah1L could suppress SKBR3 breast cancer cell invasiveness while Siah1 siRNA potently increased invasiveness in MCF-7 cells. Siah1ΔR overexpression showed less potent effect. In a previous study, it has been shown that Siah1 is directly inhibited by BAG-1 (Bcl-2-associated athanogene-1), a heat shock protein 70 (Hsp70)-associated antiapoptotic protein [12]. Additionally, BAG-1 has been shown to promote cell migration through cooperation with cytoskeletal proteins such as actin and cytokeratin [37]. Thus, Siah1 gain-of-function could reverse the latter effect. Our data are similar to a recent study which demonstrated increased proliferation, decreased apoptosis and increased invasion of Siah1 siRNA in MCF-7 breast cancer cells [38]. In that work, Wen et al. showed that the JNK and ERK signaling pathways may play an important role in the Siah1-mediated cell apoptosis and invasion, respectively. β-catenin can function as an oncogene when it is translocated to the nucleus, binds to Tcf/Lef family members and transactivates its target genes [39]. Additionally, β-catenin is involved in breast cancer formation and/or progression and may serve as a target for breast cancer therapy [40]. Since β-catenin forms a complex with a member of the Tcf/Lef family and activates the target genes, reporter assays were performed to monitor Tcf/Lef dependent gene expression in the presence or absence of Siah-1L or Siah-1. Notably, overexpressing Siah-1 and Siah1L in SKBR3 cells resulted in a reduction in the relative Tcf/Lef transcriptional activity. These findings are in line with previous reports indicating that Siah-1 and Siah1L regulate Tcf/Lef activity [20-22]. Notably, functional inhibition of Siah1 by siRNA upregulated Tcf/Lef activity in breast cancer cells, in accordance to previous reports [20,41].

The repair of DNA double-strand breaks after ionizing radiation may account for the apoptotic response and ultimate radioresinsitivity of malignant breast cancer cells, even though other mechanisms cannot be excluded. Importantly, suppression of Tcf/Lef activity, an important transcriptional factor in regulation of gene expression has been shown to occur after Siah1L overexpression [21].

Phosphorylation of H2AX seems to play a critical role in the recruitment of repair- and damage-signalling factors to the site of DNA breaks [30]. Employment of γH2AX has been extensively used to monitor the extent of DSB induction and analyse the effectiveness of novel biological therapies [31]. The number of foci correlates with the DNA damage in the nucleus and loss of γH2AX foci has been in cells within 6 hours after IR [42]. In line with this finding, we found that Siah1L prolonged the persistence of γH2AX foci even at 6 h post-IR as compared with control cells, as indicated by a higher amount of γH2AX foci in cells with stable Siah1L overexpression. Thus, both the Siah1L-induced attenuation of proliferation rate and the impaired post-IR DNA damage repair, may well have contributed to the induction of apoptosis. These findings are also in good correlation with the reduced survival of these cells, indicting potentially lethal DNA damage in Siah1L-transfected SKBR3 cells after IR. Importantly, the increased clonogenic survival of MCF-7 cells detected after silencing of Siah1 post-IR highlights the key role of Siah1 in determining response of breast cancer cells to radiotherapy.

## Conclusions

In summary, our findings show for the first time that Siah1 significantly potentiates radiation response in breast cancer. We showed that this effect is more pronounced using its splicing variant Siah1L that exhibited potentially synergistic activity in combination with radiotherapy against breast cancer cells. The possible underlying mechanisms by which Siah1 and Siah1L may decrease cell survival upon radiation exposure seem to be multifaceted. Our results suggest that gene therapeutic or pharmacological approaches enhancing Siah1 and Siah1L in tumors with absent expression of these genes could increase the therapeutic ratio for breast cancer and have important implications for the development of novel strategies in radiotherapy.

## Competing interests

The authors declare that they have no competing interests.

## Authors' contributions

HTH and AY carried out the experimental studies. EF drafted and completed the manuscript. HXA and REC participated in the design of the study and performed the statistical analysis. HTH, EF and YA participated the evaluation of analysed parameters and tumor pathological characteristics. REC conceived of the study and participated in the design and coordination as well as helped to draft the manuscript. All authors read and approved the final manuscript.

## Pre-publication history

The pre-publication history for this paper can be accessed here:

http://www.biomedcentral.com/1471-2407/10/403/prepub
